# Case of Punctured Wound of the Ascending Aorta, Fatal in Fifteen Minutes

**Published:** 1841-08

**Authors:** C. R. Gilman

**Affiliations:** Prof. Obstetrics and Diseases of Women in the College of Physicians and Surgeons, N. Y.


*Case of Punctured Wound of the Ascending Aorta, fatal in fiifteen minutes.* By C. R. Gilman, M. D., Prof. Obstetrics and Diseases of Women in the College of Physicians and Surgeons, N. Y.

*July* 17*th*, 1841. Examined, by request of the Coroner, the body of M. Riley, aged 37 years. She received last evening a stab from a sword-cane in the right breast; after the wound was inflicted she was assisted into an adjoining room and laid on the floor, where she expired in about fifteen or twenty minutes. A very small punctured wound appeared on the right breast, three and three-quarter inches inward and one inch upward from the right nipple. On introducing a probe, the wound was found to pass downward and inward for near two inches, and then to penetrate the chest, passing between the cartilages of the second and third ribs, close to the sternum. Opening the thorax, the cellular substance in the anterior mediastinum was found infiltrated with blood, and the pericardium much distended. A small punctured wound in the anterior and superior part of the pericardium was discovered after considerable search. The sac was next laid open, and found to contain a pint or more of coagulated blood—it was quite full; removing this, a large patch of ecchymosis was found upon the ascending aorta and the arch, in the midst of which a small puncture was detected. The artery was now slit up, and the wound more accurately examined from within. It was V shaped each side, about a line in length, situated about half an inch above the valve, and equi-distant between the orifices of the two coronary arteries. There was no other wound of the internal membrane of the artery or the heart. The instrument would seem to have penetrated but not passed through the organ.

*Remarks.*—The points of interest in this case are the suddenness of death, and the peculiar V shape of the wound in the aorta answering very exactly to the size and triangular shape of the instrument with which the injury was inflicted. As to the suddenness of death under such circumstances, the majority of authors agree with Margagni (*Ep.* 69, *sec.* 5,) in ascribing it, not to the loss of blood, but to the obstruction offered to the circulation by the blood having accumulated in the pericardium. This, though the common explanation, is, I think, incorrect, or at least incomplete; for there are not wanting many cases where the heart having ruptured, two or three pounds of blood have been found in the pericardium, and yet life has continued for several hours. Olmi, of Florence, gives one; the rupture was an inch long, the patient lived "till next day," "a quantity" of grumous blood occupied the pericardium. Hufeland's Journal gives a case, where the rupture was half an inch internally, larger externally; the man lived three days, two or three pounds of blood were in the pericardium. A similar case is given by Dezeimeris in *L'Experience*, 1839. It is not, then, merely to the presence of a quantity of blood in the pericardium, that death is owing. The other modifying circumstance, is the rapidity with which the blood is effused. Hence death takes place less rapidly in ruptures where the heart's tissue is sound or merely softened, and where the opening is usually made in the direction of the fibres which are merely separated, than in cases where there is an opening with loss of substance from ulceration—the latter almost always proving instantly fatal. In wounds of the heart, those which bisect the two layers of fibres are suddenly fatal, while in those which, though they bisect one layer, only push apart the fibres of the other, life continues longer: this must be attributed to the manner in which contraction of the fibres opens the one wound and closes the other. It is in wounds of the aorta within the pericardium that hæmorrhage is most free and death most sudden: the blood passes much more rapidly through the opening in the thin coat of the vessel than where it has to make a devious way through the thick paries of the heart. Even these, however, are not always suddenly fatal. In the Journal de Medicine, vol. 40, p. 435, a case is given where the patient lived to the sixth day; the aorta was penetrated near its origin. Leroux gives a case where death did not occur till the eleventh day, though the aorta and the right auricle were both wounded. This is the more remarkable, as wounds of the auricle are generally more quickly fatal than those of the ventricle. Pelletan, in his Clinique Chirurgicale, t. 1, p. 92, gives a case not fatal till after two months: the wound of the aorta was near the crura of the diaphragm. A case of rupture of the aorta is reported in the Medico-Chirurgical, April, 1840, p. 612, where the patient lived ten hours. The opening however, was not direct, the inner coat of the vessel gave way immediately behind the semi-lunar valve and close to the mouth of the posterior coronary artery, while the outer coat burst further up, where the arch emerges from behind the pulmonary artery.

The shape of the wound through the aorta in this case, is worthy of notice. The wound through the skin had no peculiarity to distinguish the instrument by which it was inflicted —it was very small and nearly circular—but that in the aorta was triangular or V shaped, and exactly answered to the shape of the weapon. This shows the importance of examining for the shape of the wound, not in the skin only, but internally, particularly when the instrument has passed through a dense fibrous membrane.—*N. Y. Med. Gaz.*

